# A123 MINIMALLY INVASIVE ENDOSCOPIC RESECTION TECHNIQUE PERFORMANCE FOR PERI-APPENDICEAL COLORECTAL NEOPLASIA: A SYSTEMATIC REVIEW AND META-ANALYSIS

**DOI:** 10.1093/jcag/gwab049.122

**Published:** 2022-02-21

**Authors:** A A Arif, K Donaldson, H Qian, E Lam, N C Shahidi

**Affiliations:** 1 Medicine, The University of British Columbia Faculty of Medicine, Vancouver, BC, Canada; 2 Centre for Health Evaluation and Outcome Sciences, Vancouver, BC, Canada; 3 St. Paul’s hospital, Division of Gastroenterology, Vancouver, BC, Canada

## Abstract

**Background:**

Minimally invasive endoscopic resection techniques, including endoscopic mucosal resection (EMR), endoscopic submucosal dissection (ESD) and endoscopic full-thickness resection (EFTR) have revolutionized the management of peri-appendiceal colorectal neoplasia. However, questions remain about their comparative performance.

**Aims:**

We sought to evaluate the performance of EMR, ESD and EFTR for peri-appendiceal colorectal neoplasia.

**Methods:**

Two authors independently searched MEDLINE, EMBASE and Cochrane Libraries (Jan 2000 – Aug 2021) for citations evaluating the performance of endoscopic resection techniques (EMR, ESD, EFTR) for peri-appendiceal colorectal neoplasia (defined as those involving or in close proximity to the appendiceal orifice). The incidence rates and 95% confidence intervals (95% CI) of technical success (complete removal of all neoplastic tissue at index procedure), clinically significant post-endoscopic resection bleeding (CSPEB), delayed perforation, recurrence and referral to surgery were assessed using random-effects modelling.

**Results:**

12 studies were included in the analysis (479 patients: 185 EMR, 171 ESD, 123 EFTR). Technical success was achieved in 93.5% (95% CI 90.9%-95.4%, EMR 93.5%, ESD 94.1%, EFTR 92.7%). Clinically significant post-endoscopic resection bleeding occurred in 1.3% (95% CI 0.4%-4.3%, EMR 3.8%, ESD 1.2%, EFTR 0%). Delayed perforation occurred in 1.9% (95% CI 0.9%-3.9%, EMR 0%, ESD 2.4%, EFTR 2.4%). Recurrence occurred in 5.7% (95% CI 2.3%-13.8%, EMR 14.3%, ESD 0.2%, EFTR 12.2–14.3%). Referral to surgery occurred in 9.0% (95% CI 6.7%-12.0%, EMR 8.1%, ESD 9.5%, EFTR 9.8%).

**Conclusions:**

Minimally invasive endoscopic resection techniques including EMR, ESD and EFTR demonstrate high frequencies of technical success with comparable adverse event profiles. They should now be viewed as first-line therapeutic modalities for the management of peri-appendiceal colorectal neoplasia.

**Funding Agencies:**

None

